# Evolving landscape of methicillin-resistant *Staphylococcus pseudintermedius*: the emergence of new epidemic waves across Europe, Asia and North America

**DOI:** 10.1093/jac/dkaf340

**Published:** 2025-09-27

**Authors:** Lillian Rose Giarratana, Mattia Pirolo, Franz-Ferdinand Roch, Beate Conrady, Luca Guardabassi

**Affiliations:** Department of Veterinary and Animal Sciences, University of Copenhagen, Frederiksberg C 1870, Denmark; Department of Veterinary and Animal Sciences, University of Copenhagen, Frederiksberg C 1870, Denmark; Clinical Department for Farm Animals and Food System Science, Centre for Food Science and Veterinary Public Health, University of Veterinary Medicine, Vienna 1210, Austria; Department of Veterinary and Animal Sciences, University of Copenhagen, Frederiksberg C 1870, Denmark; Department of Veterinary and Animal Sciences, University of Copenhagen, Frederiksberg C 1870, Denmark

## Abstract

**Objective:**

This study aims to identify spatiotemporal variations in clonal diversity and antimicrobial resistance of methicillin-resistant *Staphylococcus pseudintermedius* (MRSP).

**Materials and methods:**

A PubMed search (June 2016–December 2023), combined with data from PubMLST and a previous review (September 2007–May 2016), identified 2654 isolates. Multinomial logistic regression (MLR) and Bayesian regression models (BRMs) were used to assess associations between clonal complexes (CCs) and variables (sample type, continent and period), with MLR results feeding into the BRM.

**Results:**

A shift in MRSP clonal structure was observed after 2013. A decline in the prevalence of historically dominant lineages, such as CC71 in Europe, CC68 in North America and CC45 in Asia, coincided with the global emergence of CC551, as well as CC556 and CC1431 in North America, and CC363 and CC1631 in Asia. Resistance to non-β-lactams increased in North America, particularly for chloramphenicol (6%–59%), remained largely stable in Europe except for tetracycline, and decreased in Asia. Striking differences among CCs were observed, with CC71 exhibiting the highest resistance rates and a greater likelihood of being isolated from clinical samples.

**Discussion:**

The observed associations between specific CCs and resistance patterns provide valuable insights into the factors driving these epidemiological changes, including regional antimicrobial use patterns (e.g. chloramphenicol usage in North America) and potential fitness advantages of emerging lineages. These dynamics parallel those seen in methicillin-resistant *Staphylococcus aureus*.

**Conclusion:**

The evolving MRSP landscape highlights the need for sustained global surveillance to monitor clonal diversity, antimicrobial use and resistance trends.

## Introduction

*Staphylococcus pseudintermedius*, previously named *Staphylococcus intermedius*, is part of the normal microbiota of dogs, yet is also the most common aetiologic agent of canine pyoderma, ear, postoperative and urinary tract infections (UTIs).^1^ While historically associated with infections in dogs, there is growing evidence of its ability to infect other hosts, including cats and humans.^2,3^ The simultaneous escalation of multidrug-resistant strains, particularly methicillin-resistant *S. pseudintermedius* (MRSP), presents therapeutic challenges due to the shortage of effective veterinary antimicrobials. Hence, surveillance is crucial for tracking the prevalence of MRSP strains and their resistance profiles. However, surveillance programmes monitoring antimicrobial resistance (AMR) in pathogens affecting companion animals are limited to only a few countries. In Europe, the mean prevalence of methicillin resistance among clinical isolates from dogs and cats is estimated at 5.8%, with significant variability between countries, reaching over 40% in some studies.^4^

Multilocus sequence typing (MLST) has been instrumental in revealing the population structure and epidemiological trends of MRSP on a global scale. At the time MRSP emerged approximately 20 years ago, two major clonal lineages disseminated in Europe (ST71-SCC*mec* II–III) and North America (ST68-SCC*mec* V), respectively.^5^ In 2016, a systematic review based on MLST data from the scientific literature depicted the population structure, geographical distribution and antibiotic resistance patterns of the main MRSP lineages at that time.^6^ Here, we provide an updated review coupled with a spatial–temporal meta-analysis to track the evolution of MRSP over the last decade. Our meta-analysis provides important insights into the geographic expansion of new MRSP lineages, unveiling noteworthy changes in population structure and AMR patterns.

## Materials and methods

### Search strategy and study selection

**Figure 1.**
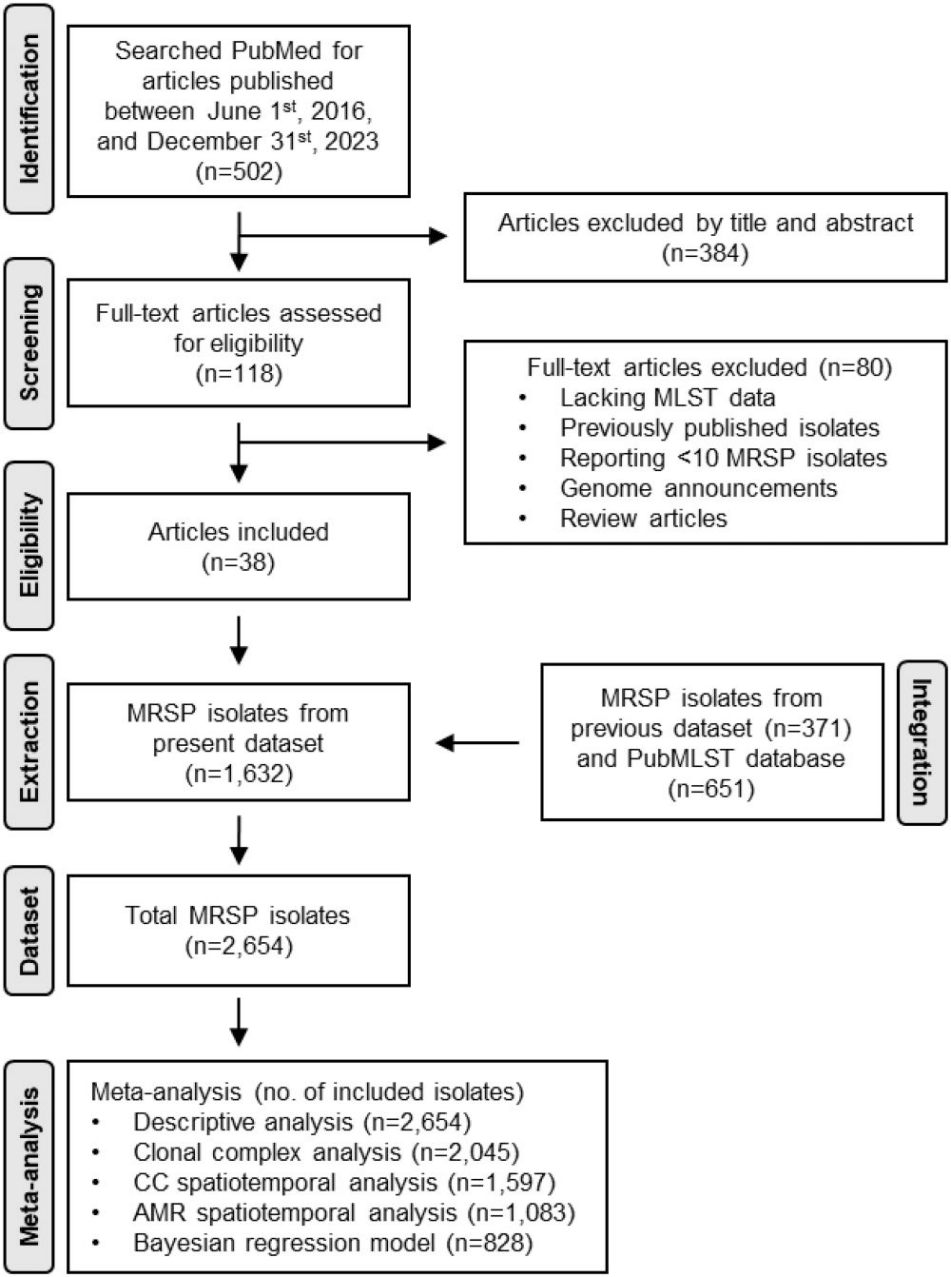
Flowchart of the literature search, studies selection, data extraction and isolates included in each meta-analysis.

A literature search was conducted within the PubMed database using the term ‘*Staphylococcus pseudintermedius*’ covering the period from 1 June 2016 to 31 December 2023. Figure 1 illustrates a flowchart delineating the selection strategy. Exclusion criteria were (i) studies not reporting MLST data, (ii) studies based on previously published MRSP isolates, (iii) studies reporting less than 10 MRSP isolates, (iv) in languages other than English, (v) genome announcements and (vi) reviews. Out of 502 records identified by abstract screening, 118 papers were selected for full-text examination, and 46 eligible papers were selected based on the above exclusion criteria.

### Data extraction

Individual isolate data extraction was executed and integrated with our pre-existing dataset,^6^ supplemented by additional isolates from the PubMLST database (accessed on 16 January 2024). We included only isolates characterized using the current MLST-7 scheme,^7^ while those typed with the earlier MSLT-5 scheme were excluded. Extracted data included sequence type (ST), host, sampling location and year of isolation. For isolates reported in scientific publications, antimicrobial susceptibility profiles were also retrieved when available. To avoid duplicate MRSP isolates, we reviewed methods sections for studies lacking isolate IDs to assess whether isolates came from unique sources. In longitudinal studies, only the first isolate per subject was included, except in cases where different STs were identified. We also cross-referenced studies by the same groups using metadata such as isolation year and country. Isolates of uncertain uniqueness were conservatively excluded from the meta-analysis. In the analysis of susceptibility data, we adhered to the criteria utilized in the original papers to categorize isolates as resistant or susceptible. Isolates categorized as intermediate, currently renamed as ‘susceptible dose-dependent’ by CLSI,^8^ were reclassified as susceptible for the purposes of this study. The dataset consisted of 2654 entries representing individual MRSP isolates from 46 studies or the PubMLST database. Each isolate was assigned a unique identifier (ranging from ID0001 to ID2654). The dataset included detailed information such as ST, clonal complex (CC), country and continent of origin, year of isolation, host, infection status, isolation site, sample type and antimicrobial susceptibility testing results for chloramphenicol, clindamycin, enrofloxacin, gentamicin, trimethoprim/sulfamethoxazole and tetracycline. These antimicrobials were chosen due to their inclusion in most studies and their relevance for treatment of MRSP infections.

CC assignation was performed based on MLST allelic profiles through two consecutive rounds of ST clustering based on the eBURST algorithm,^9^ as implemented in the *S. pseudintermedius* PubMLST database. Briefly, in the first round, all STs were analysed to identify the main CCs. STs not assigned to a CC in the first round were then re-analysed in a second round to capture additional potential groupings. For each round, STs were grouped into a CC if their MLST profiles matched at five or six loci with at least one other ST in the cluster (i.e. single and double locus variants, respectively).^10^

### Statistical analysis

The meta-analysis primarily focused on three aspects: (i) associations between CCs and independent variables (i.e. sample type, continent and period); (ii) the predicted probability of detecting each CC, based on the significant independent variables identified in the analysis from (i); and (iii) associations between resistance levels and CCs, sample type, continent and period. Sample type was categorized into five categories: colonization, skin and soft tissue infection (SSTI), wound infection, UTI and otitis. To assess the associations between CCs and independent variables—specifically sample type, continent and period—we focused on dog isolates, as they constituted most of the dataset. STs that could not be grouped into CCs were excluded from the analysis. Additionally, CCs representing fewer than 10% of cases in the largest CC group were excluded to maintain model stability. For temporal analysis, isolates were categorized based on collection date and classified as collected before or after 2013. This year was subjectively chosen to balance the number of isolates before (*n* = 1016) and after (*n* = 1452), while 186 isolates were unassigned due to ambiguous records. Samples collected during 2013 were arbitrarily included in the ‘before 2013’ group to allow for binary comparison.

We employed multinomial logistic regression (MLR) and Bayesian regression models (BRMs) to determine associations between CCs and the independent variables. Final model outcomes were obtained from BRM, as this approach allows for the inclusion of studies as random factors, enhancing the model’s ability to account for variability across studies. The results from MLR were used as prior inputs to inform the BRM. BRMs with different levels of complexity were tested (Table S1 is available as Supplementary data at *JAC* Online), ending in the simplest model, due to the low sample size, subsequently resulting in weak model performance. We ran the final model with four chains, each with 10 000 iterations, using 5000 iterations as warmup (Table S2).

R-hat and effective sample size (ESS) values were analysed due to the integrated resampling procedure in this study, as well as to confirm the reliability of parameter estimates. We evaluated model fit using leave-one-out cross-validation (LOO-CV) and compared models with the LOO information criterion (LOOIC). Residual diagnostics were performed to detect any patterns indicative of model misspecification. The sensitivity analysis identified and described influential cases and influential studies, to highlight their potential impact on the model’s results, without removing them, thereby preserving the integrity of the analysis (Figure S1). Model coefficients were converted to odds ratios and probabilities. For model validation, we examined R-hat and ESS values (Table S2), distribution and trace plots ensuring all values remained below 1.05 (Figure S2) and performed posterior predictive checks, pareto-k diagnostics and a sensitivity analysis (Figure S1). The references in the model were isolates from ‘colonization’ and CC71, as most prevalent CC. The reason for choosing a Bayesian approach over a frequentist approach was the possibility to include random factors such as the study ID, which we considered as important, since we used data from many different sources.

Posterior predictive checks were carried out to compare observed data with simulated data from the model. MLR was conducted using the multinom function from the nnet package (v7.3-19),^11^ with model comparisons made via Akaike’s information criterion (AIC) and likelihood ratio tests (LRTs). All statistical analyses were conducted using R (v 4.4.1).^12^

We also analysed temporal changes in AMR patterns. For this analysis, all hosts and sample types were considered. First, we calculated differences between resistance levels over time by Fisher’s exact test and corrected *P* values using the Benjamini-Hochberg method. Then, the associations between resistance levels and CCs were analysed using binomial logistic regressions for each antimicrobial drug, using ‘study ID’ as a random factor. Only CCs with at least five resistant and susceptible isolates, or those comprising at least 5% of the isolates resistant to the drug, were included (contingency tables of the used dataset are presented in Tables S3–S8). This relatively inclusive threshold was chosen to allow representation of less prevalent CCs while still ensuring sufficient data for reliable modelling. All binomial regression models were validated (Tables S3–S8), examined for influential cases (i.e. outliers from observation index) (Figure S3) and subjected to a sensitivity analysis (Figure S4). For all comparison, CC71 was used as the reference CC. In resistance levels analysis, the glmer function from the lme4 package (v1.1-35.4)^13^ was utilized for binomial logistic regression, with optimization controlled by the BOBYQA algorithm. Residual diagnostics were conducted using the DHARMa package (v0.4.6),^14^ and overdispersion was assessed using Pearson’s chi^2^.

## Results

### Numbers, origins, STs and CC clustering of MRSP isolates

A total of 46 studies were selected for the meta-analysis (Table S9), including 38 articles from our literature review and eight articles from our previous dataset, yielding a mean of 44 MRSP isolates per study (range 10–174). The resulting MRSP database comprised 2654 MRSP isolates (Table S10), including those identified from the literature review (*n* = 1632), our prior review (*n* = 371)^6^ and the PubMLST database (*n* = 651). Isolates were predominantly originating from dogs (*n* = 2163), followed by humans (*n* = 82), environmental samples (*n* = 72) and cats (*n* = 56), while host origin was not specified for 281 isolates. Among isolates with known origin (*n* = 2313; 87.1%), the majority derived from clinical specimens (*n* = 2014), with only few cases of colonization (*n* = 227). Among the clinical isolates, 38.5% were attributed to SSTIs, 10.7% to wound infections, 9.3% to otitis and 3.3% to UTIs. Isolates from Europe (41.6%), Asia (23.8%) and North America (20.5%) collectively accounted for more than 85.0% of the isolates included in the database (2280 isolates in total), followed by Oceania (7.6%), South America (5.9%) and Africa (0.6%) (Table S11). The MRSP database comprised 754 STs. Of these, 283 (*n* = 1739 isolates) and 97 (*n* = 306 isolates) STs clustered in 18 CCs over the two iterative rounds of eBURST analysis (Figure S5), while 374 STs (*n* = 609 isolates) were not assigned to any CCs.

**Figure 2.**
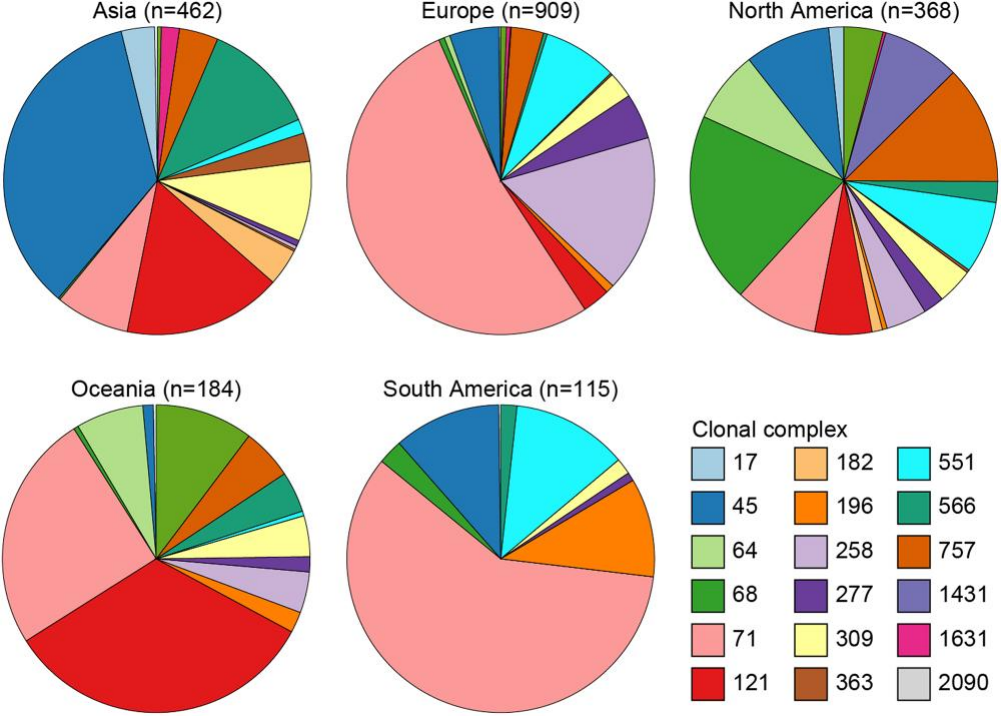
Distribution of the CCs in each continent. Eight CCs were present on all continents, and none were continent specific. North America showed the highest CC diversity (18 CCs), while CC71 was highly prevalent in Europe and South America, and CC45 in Asia. Africa was excluded due to low sample size.

The distribution of the 18 CCs across continents is shown in Figure 2. Africa was excluded from the analysis as only seven isolates clustered into a CC. Among the remaining five continents, eight CCs (CC45, CC68, CC71, CC196, CC277, CC309, CC551 and CC566) were detected in all five continents, while four CCs (CC121, CC258, CC757 and CC2090) were reported in four continents. Notably, no CC was confined to a single continent. All 18 CCs were identified in North America, with CC68 (20.1%) and CC757 (12.5%) being the most prevalent. In Europe and Asia, 17 and 16 CCs were identified, respectively. CC71 (52.7%) and CC258 (16.5%) were predominant in Europe, while CC45 (35.1%), CC121 (16.7%) and CC566 (12.1%) were the most abundant in Asia. In Oceania, 13 CCs were detected, with CC121 (33.2%) and CC71 (25.0%) being the most common. Finally, eight CCs were identified in South America, where CC71 dominated (59.1%).

### Spatiotemporal analyses

**Figure 3.**
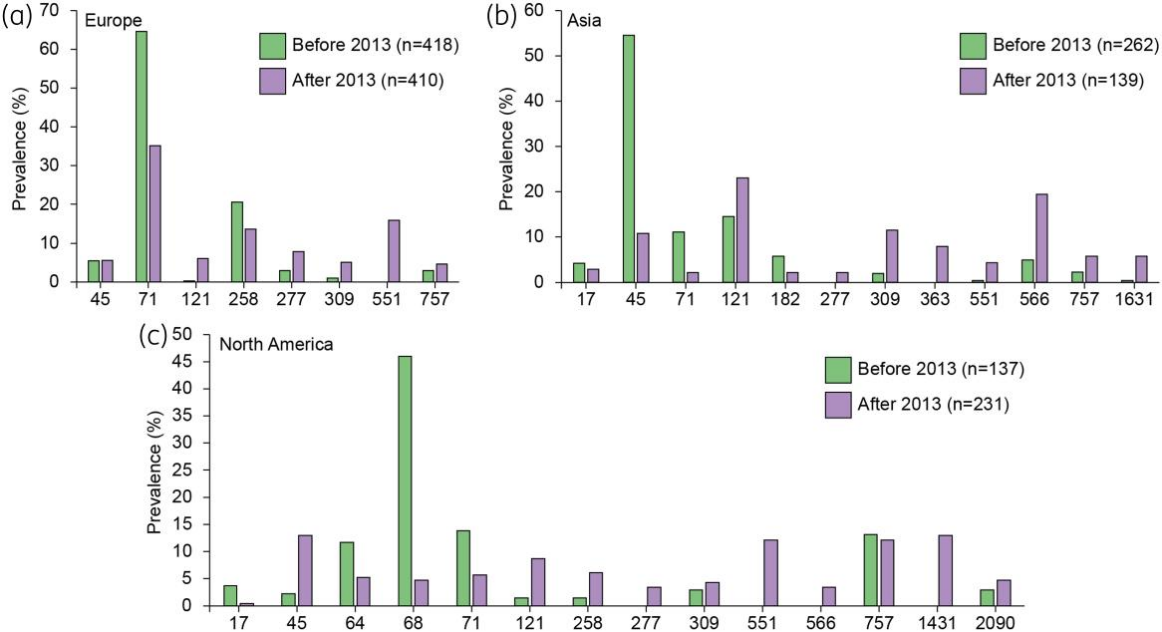
Distribution of the main CCs among Europe (a), Asia (b) and North America (c) before and after 2013. CC71 and CC45 declined across all regions, while CC551 and emerging lineages such as CC309, CC566 and CC1431 increased after 2013. Only CCs with a prevalence of >2% are illustrated.

Temporal analysis of CC distribution was limited to Europe (*n* = 828 isolates), Asia (*n* = 401) and North America (*n* = 368). This limitation was necessitated by the scarcity of isolates from Africa (*n* = 13) and the relatively low number of isolates from Oceania and South America that were available before 2013. Specifically, only 8 out of 153 isolates from Oceania and 27 out of 115 isolates from South America were obtained before 2013 and could be assigned to a defined CC. Before 2013, CC71 (64.6%) and CC258 (20.6%) were the predominant lineages circulating in Europe, and CC45 (54.6%) was the most prevalent in Asia, followed by CC121 (14.5%) and CC71 (11.1%), while CC68 (46.0%), CC71 (13.9%) and CC757 (13.1%) were the dominant lineages in North America (Figure 3). After 2013, a significant shift in MRSP lineages was observed across all three continents. The prevalence of CC71 declined in all regions, though it remained the most prevalent in Europe (35.1%), alongside CC258, which also showed a slight decline (13.7%). In Asia, CC45 and CC71 decreased to 10.8% and 2.2%, respectively, while the prevalence of CC566 (19.4%) and CC309 (11.5%) increased. In North America, CC68 dropped to 4.8%, while CC45 (13.0%), CC1431 (13.0%), CC551 (12.1%) and CC121 (8.7%) rose. Additionally, new lineages that were previously undetected emerged in one or more continents, including CC551 across all continents, CC363 and CC1631 in Asia and CC556 and CC1431 in North America (Figure 3).

Spatiotemporal analysis of AMR profiles for Europe, Asia and North America included 1083 isolates with data for at least one of the selected antimicrobials (Table 1), namely gentamicin (*n* = 1033), tetracycline (*n* = 1020), chloramphenicol (*n* = 682), clindamycin (*n* = 828), trimethoprim/sulfamethoxazole (*n* = 682) and enrofloxacin (*n* = 557). In Europe, resistance rates of MRSP isolates remained stable over time, except for tetracycline resistance, which increased from 60.4% before 2013 to 71.0% after 2013 (*P*-adj = 0.03). In Asia, resistance levels to gentamicin, clindamycin and tetracycline decreased after 2013 (*P*-adj < 0.05). In North America, chloramphenicol resistance showed a significant increase, from 6.4% before 2013 to 58.7% after 2013 (*P*-adj < 0.001). Resistance to gentamicin and tetracycline also increased significantly (*P*-adj < 0.05), while the frequency of trimethoprim/sulfamethoxazole resistance decreased during the same period (*P*-adj = 0.03). Only minor differences were observed in the resistance levels of the major CCs before and after 2013 (Table S12).

**Table 1.** Antimicrobial resistance level (%) of MRSP isolates in Europe, Asia and North America before and after 2013

Antimicrobial	Europe		Asia		North America	
	Before 2013	After 2013	Before 2013	After 2013	Before 2013	After 2013
GEN	59.4 (189/318)	67.5 (206/305)	**96.7 (118/122)**	**59.7 (37/62)**	**76.6 (108/141)**	**90.6 (77/85)**
CLI	89.1 (164/184)	85.8 (194/226)	**97.5 (119/122)**	**61.5 (24/39)**	82.3 (116/141)	84.5 (98/116)
TET	**60.4 (198/328)**	**71 (206/290)**	**99.2 (121/122)**	**91.9 (57/62)**	**77.3 (109/141)**	**90.9 (70/77)**
CHL	20.7 (46/222)	24.8 (62/250)	65.3 (79/121)	54.8 (34/62)	**6.4 (9/141)**	**58.7 (27/46)**
SXT	73.6 (128/174)	76.6 (147/192)	80 (8/10)	69.4 (43/62)	**88.7 (125/141)**	**76.7 (79/103)**
ENR	67.7 (170/251)	67.9 (163/240)	90 (9/10)	78.3 (18/23)	NA	57.6 (19/33)

Significant differences in the frequency of resistance between isolates collected before and after 2013 are highlighted in bold.

CHL, chloramphenicol; CLI, clindamycin; ENR, enrofloxacin; GEN, gentamicin; SXT, trimethoprim/sulfamethoxazole; TET, tetracycline; NA, not available.

### Associations between CCs and sample type

To describe the associations between CCs and different sample types, we focused only on isolates from dogs, which are the most prevalent hosts, and from the most common sample types/conditions (i.e. ‘colonization’, ‘otitis’, ‘SSTI’, ‘UTI’ and ‘wound infection’). Further, only CCs that represents at least 10% of the total isolates were considered (*n* = 9). After filtering, a total of 828 isolates from 36 studies were included in the BRMs.

**Figure 4.**
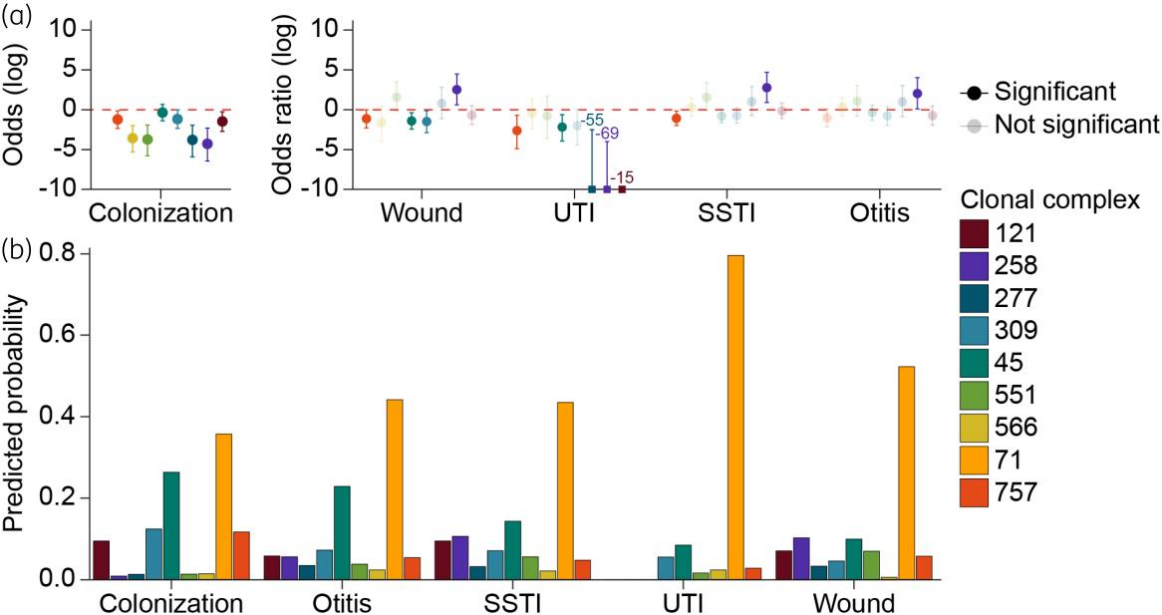
Results of the BRM. (a) Association between individual CC and colonization and sample types. Compared with CC71, odds for CC258 increase in wound, SSTI and otitis, while CC45, CC309 and CC757 are more associated with colonization. Significant odds ratios are shown as filled colours. (b) Predicted probability of detecting individual CC in colonization and sample types, showing CC71 as the most likely lineage across all sample types. SSTI, soft tissue infection; UTI, urinary tract infection.

Overall, CC71 was the most prevalent isolate in all sample types (used as reference in the BRM). In the reference sample ‘colonization’, the odds of having another CC over CC71 ranged from −4.28 (CC258/CC71) to −0.36 (CC45/CC71). Among clinical samples, the odds from other CCs over CC71 shifted significantly depending on the disease condition. Compared with colonization, the odds of CC757/CC71 were significantly lower in wound infections (−1.13), UTI (−2.62) and SSTI (−1.08) (Figure 4a and Table S2). The same applied to CC45/CC71 in wound infections (−1.41) and UTI (−2.18) and CC309/CC71 in wound infections (−1.48). The odds of CC258/CC71, on the other hand, were significantly increased in wound infections (2.52), SSTI (2.76) and otitis (2.03). The strongly negative values for CC121, CC258 and CC277 in UTI were caused by the absence of these CCs in isolates from UTI.

The predicted probabilities for each CC and sample type indicated that CC71 was the most probable CC to isolate from all sample types, but even higher in infections (43.4%–79.5%) compared with colonization (35.6%). CC45, CC309 and CC757 showed higher chances of being isolated from colonization samples than from other conditions, while CC258, CC277 and CC551 had higher chances to be isolated from infections (Figure 4b). However, the BRM could not resolve for very important additional factors, such as the spatiotemporal factors, namely continent and isolation year (before or after 2013). A MLR revealed that both these factors significantly influenced the predicted probabilities for each CC (Figure S6). This likely reflects the fact that most of the isolates originated from Europe (*n* = 495) and were collected after 2013 (*n* = 450).

### Association between resistance levels and CCs

**Figure 5.**
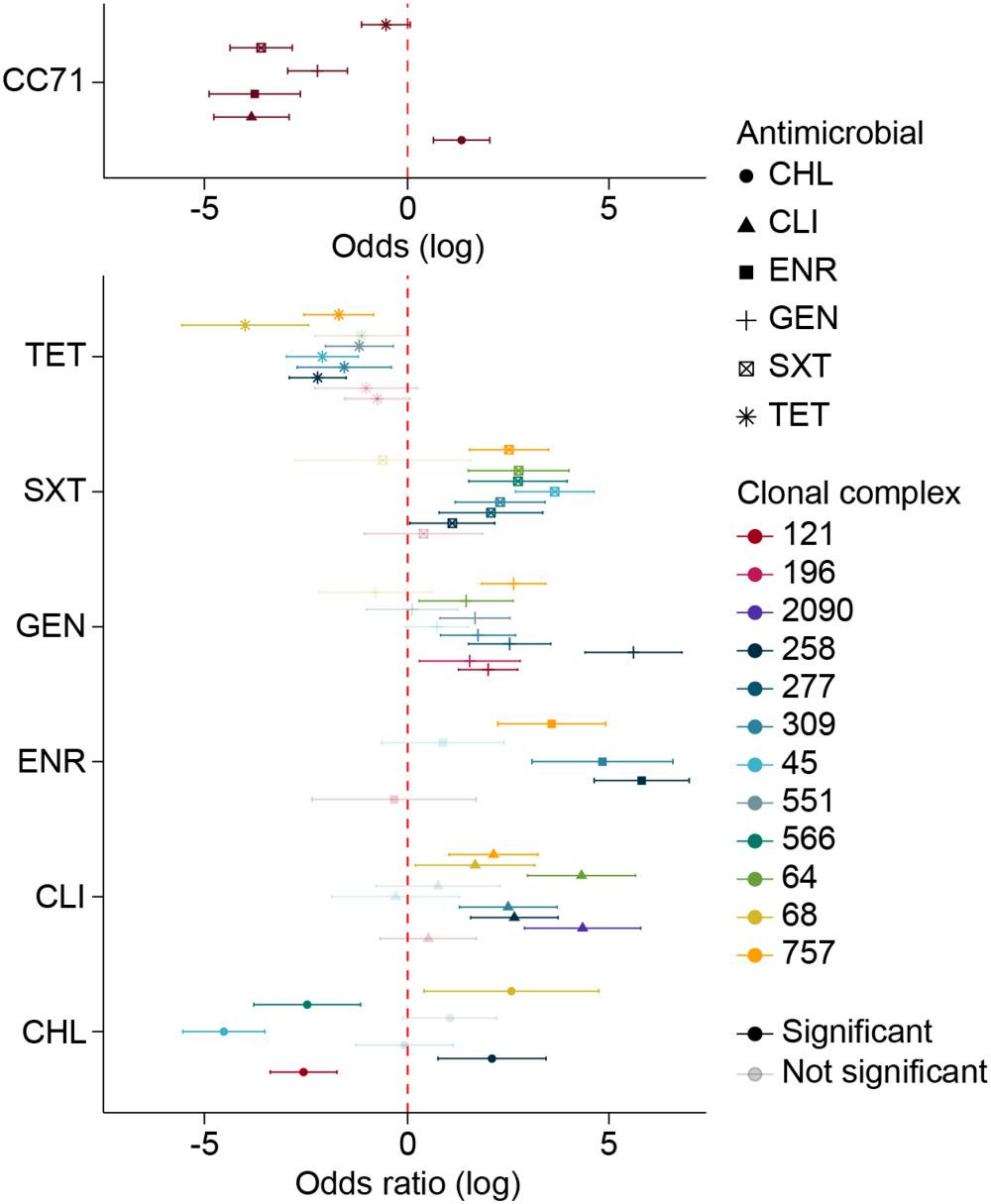
Association between resistance levels and CCs, stratified by antimicrobials. CC71 showed higher resistance to clindamycin (CLI), enrofloxacin (ENR), gentamicin (GEN) and trimethoprim/sulfamethoxazole (SXT). Tetracycline (TET) resistance was significantly higher in other six CCs, while chloramphenicol (CHL) resistance varied by CC. Significant odds ratios are shown as filled colours.

Overall, resistance to non-β-lactams was more prevalent in CC71 than in other CCs except for tetracycline and chloramphenicol (Figure 5). Six of the nine CCs tested (CC258, CC309, CC45, CC551, CC68 and CC757) had significantly higher tetracycline resistance rates than CC71 (Figure 5 and Table S5). Chloramphenicol resistance was significantly higher in CC45, CC121 and CC566 and lower in CC68 and CC258 compared with CC71 (Figure 5 and Table S6).

## Discussion

Our analysis of 2654 MRSP isolates across 46 studies revealed significant trends in the distribution of CCs and AMR profiles globally. The results highlight key spatiotemporal variations and specific associations between lineages, resistance profiles and sample types. Notably, we found high variability in the distribution of CCs across continents, implying regional differences in the clonal dynamics of MRSP. CC71 and CC258 were confirmed to remain dominant in Europe, despite a decline observed after 2013. In contrast, Asia and North America exhibited a more diverse set of circulating CCs, with notable shifts in the predominance of certain lineages over time.

The spatiotemporal analysis revealed that MRSP lineages have undergone significant changes in their distribution after 2013. In Europe, CC71 and CC258 remained prevalent, although their frequencies declined over time, while new lineages such as CC551 emerged across multiple continents, as previously reported in Northern Europe and France.^15–17^ In Asia, a marked reduction in CC45 and CC71 prevalence coincided with an increase in CC566 and CC309. Similarly, in North America, while CC68 declined, CC45, CC1431, CC551 and CC121 emerged as more dominant lineages. This trend is consistent with the findings by Phophi *et al*.^18^ in the USA. Altogether these findings are consistent with the global emergence of new lineages, especially CC551 and CC309, that have partly replaced the previously prevalent CCs, such as CC71, CC45 and CC68 in Europe, Asia and North America, respectively. Recent studies published after the completion of our literature review confirm our findings, showing a decline in CC71 in Sweden with new clones such as CC258, ST265 and CC551 emerging.^19^ A rising prevalence of CC551 has been noted in Scotland and Brazil.^20,21^

The observed changes in clonal distribution resemble the epidemiological evolution observed in methicillin-resistant *Staphylococcus aureus* (MRSA), characterized by successive waves of new epidemiologically successful clones gradually replacing previously prevalent ones.^22^ These waves likely result from a combination of selective pressures, including temporal or geographical variations in antimicrobial use, host interactions and the adaptive advantages of emerging clones. Understanding lineage-specific factors driving these variations is crucial. For MRSA, it has been hypothesized that SCC*mec* IV contributed to the rise and success of community-acquired lineages in the 1990s due to its smaller size, lower fitness and presumably higher mobility compared with SCC*mec* I–III associated with the earlier waves of hospital-acquired lineages.^22^ A similar pattern may have occurred in the evolution of MRSP since the first globally dominant lineage (CC71), although there is no evidence that this pattern is linked to SCC*mec* size. Another parallel between MRSP CC71 and the archaic MRSA clones that first emerged in human medicine is the association with hospitalization, which induced Kasai *et al*.^23^ to designate CC71 as a healthcare-associated MRSP clone.

Differences in AMR patterns among CCs were striking. CC71 exhibited a higher resistance to non-β-lactams, in line with our previous review indicating that this CC is strongly associated with multidrug resistance.^6^ Interestingly, CC258, CC309 and CC551 displayed higher tetracycline resistance, while CC45 and CC121 showed elevated chloramphenicol resistance compared with CC71. These observations highlight the variability in resistance profiles among CCs, with certain lineages displaying distinct patterns likely influenced by their geographic distribution and the antimicrobial pressures in those regions. The shifts in CC distribution observed after 2013 may be driven by factors such as changes in antimicrobial usage, including increased reliance on second-line agents such as chloramphenicol and doxycycline for treating MRSP infections. The significant rise in chloramphenicol resistance in North America coincided with the spread of lineages that often display this resistance phenotype, such as CC45, CC121 and CC566. In the USA, chloramphenicol is available for systemic therapy in dogs, including one FDA-approved oral formulation (Viceton^®^) as well as various compounded formulations provided by licensed pharmacies. In contrast, in Europe, chloramphenicol products authorized for use in dogs are generally limited to topical eye drops, whereas systemic formulations are typically unavailable due to regulatory and safety concerns. This stark difference in availability may have contributed to geographical differences in chloramphenicol resistance patterns. However, the lack of comprehensive surveillance data on antimicrobial usage in companion animals limits our ability to directly correlate patterns of antimicrobial usage with AMR trends in MRSP.

The association between CCs and sample types further underlined the potential pathogenicity and site-specific tropism of certain clonal lineages. For instance, CC71, the most frequently isolated lineage across all sample types, exhibited a higher probability of being found in clinical samples, particularly in SSTI, wound infections and otitis. On the other hand, CC45, CC309 and CC757 were more commonly isolated from colonization samples, suggesting that these lineages may be more associated with asymptomatic carriage rather than infection. This aligns with previous findings that CC71 isolates show greater adherence to canine corneocytes and an enhanced ability to produce biofilm compared with MRSP non-CC71 and methicillin-susceptible isolates.^24,25^ However, the genetic basis of these phenotypic traits cannot be explained by a difference in virulence factors.^16^ Cheung *et al*.^26^ recently discovered that CC71 is associated with the production of the PSM-*mec* peptide toxin, identical to that found in successful epidemic lineages of *S. aureus* and *Staphylococcus epidermidis*, suggesting that this virulence factor may contribute to the epidemiological success of this lineage. Additionally, the characteristic multidrug resistance phenotype of CC71 likely provides a competitive advantage, facilitating its persistence and dissemination. However, the factors driving the success of CC71 and other emerging epidemic MRSP clones remain poorly understood.

This study is not without limitations. The strong influence of ‘study ID’ as a random factor emphasizes the variability introduced by different study designs and data sources, which underscores the need for standardized methodologies in MRSP surveillance and analysis. Antimicrobial susceptibility testing methods varied across studies, and interpretative criteria were inconsistent, complicating the comparison of resistance levels. Additionally, the number of isolates per study varied widely, ranging from 10 to 174, which could introduce further bias in the analysis. The geographical distribution of isolates was skewed, with underrepresentation from regions such as Africa and Eastern Europe. This uneven sampling limits the generalizability of our findings and underscores the need for more globally representative data.

In conclusion, our study highlights the emergence of diverse MRSP lineages such as CC551 and CC309, alongside the widespread decline of CC71 and near disappearance of CC68. These shifts likely reflect selective pressures, regional antimicrobial practices and lineage-specific traits, paralleling the evolution of MRSA. The associations between specific CCs, clinical samples and AMR patterns provide valuable insights into the factors driving these epidemiological changes over the past two decades. The continuous evolution of MRSP underscores the need for ongoing surveillance to track clonal diversity and AMR trends, especially given the limited treatment options available for MRSP infections in small animal veterinary medicine.

## Supplementary Material

dkaf340_Supplementary_Data

## Supplementary data

Figures S1–S6 and Tables S1–S12 are available as Supplementary data at *JAC* Online.
