# Supplementary material for: Evolving landscape of methicillin-resistant Staphylococcus pseudintermedius: the emergence of new epidemic waves across Europe, Asia and North America
Source: J Antimicrob Chemother. 2025 Sep 27;81(1):dkaf340. doi: 10.1093/jac/dkaf340 (PMC12802899; doi:10.1093/jac/dkaf340)
Supplement: dkaf340_Supplementary_Data [file dkaf340_supplementary_data.zip › Supplementary Figures.docx]

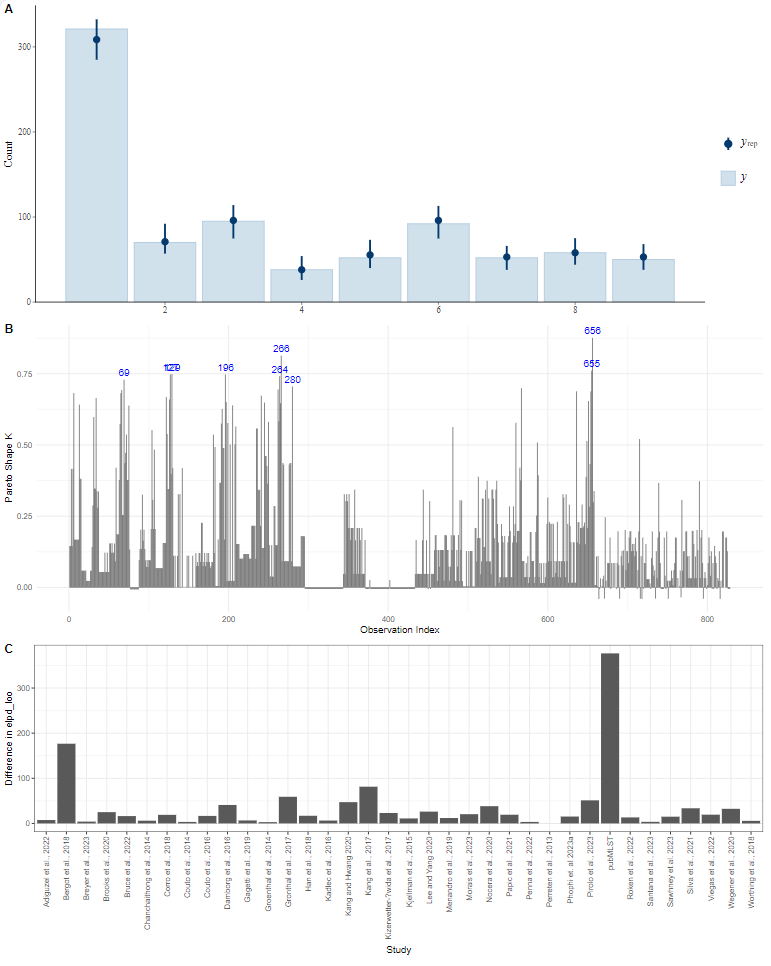


**Figure S1.** Validation of the Bayesian regression model. A) Posterior prediction check; B) Influential cases (isolates); C) Sensitivity analysis (influential studies).


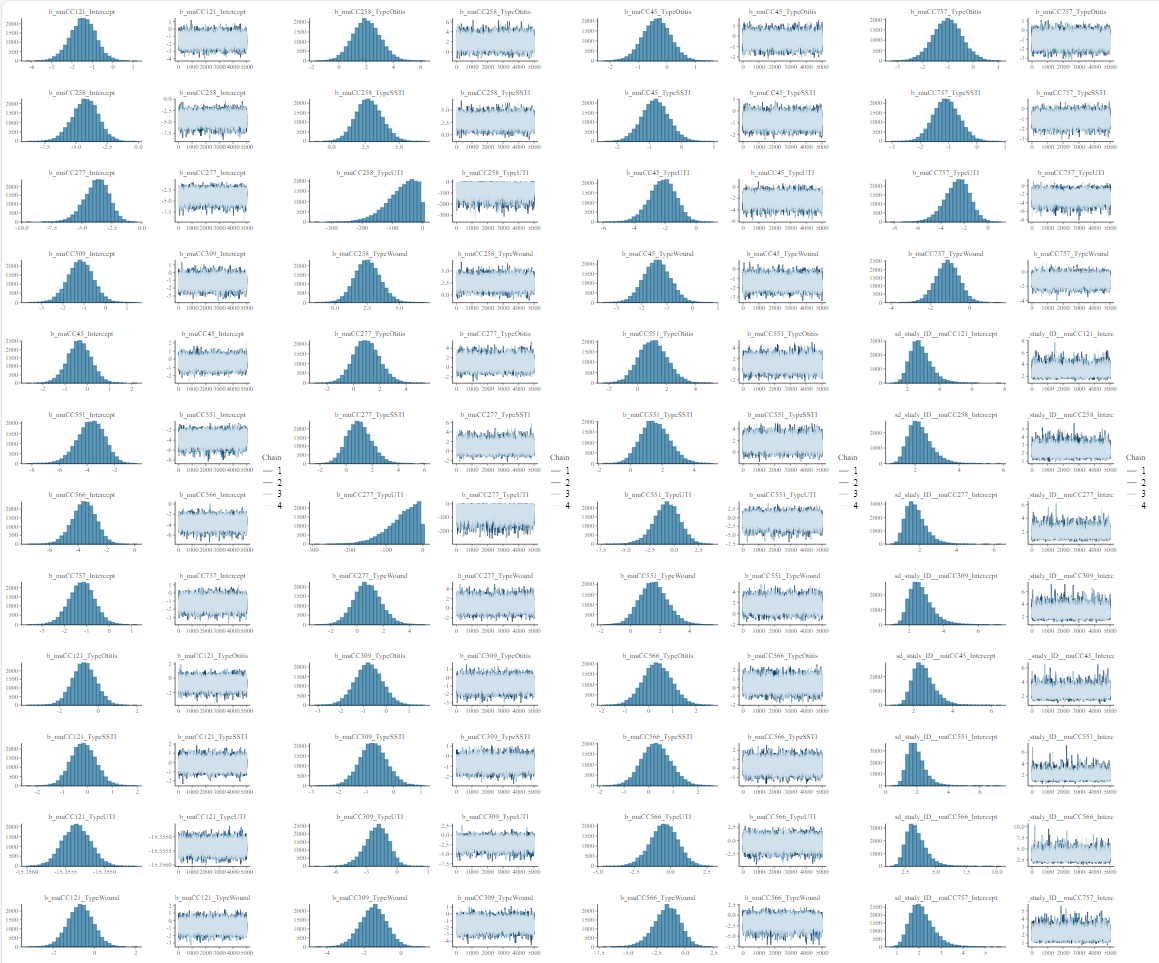


**Figure S2.** Distribution and Trace Plots stratified by CC and Sample type and Intercept. Some distributions, such as CC258 associated with UTI, appear skewed and should be interpreted with caution.
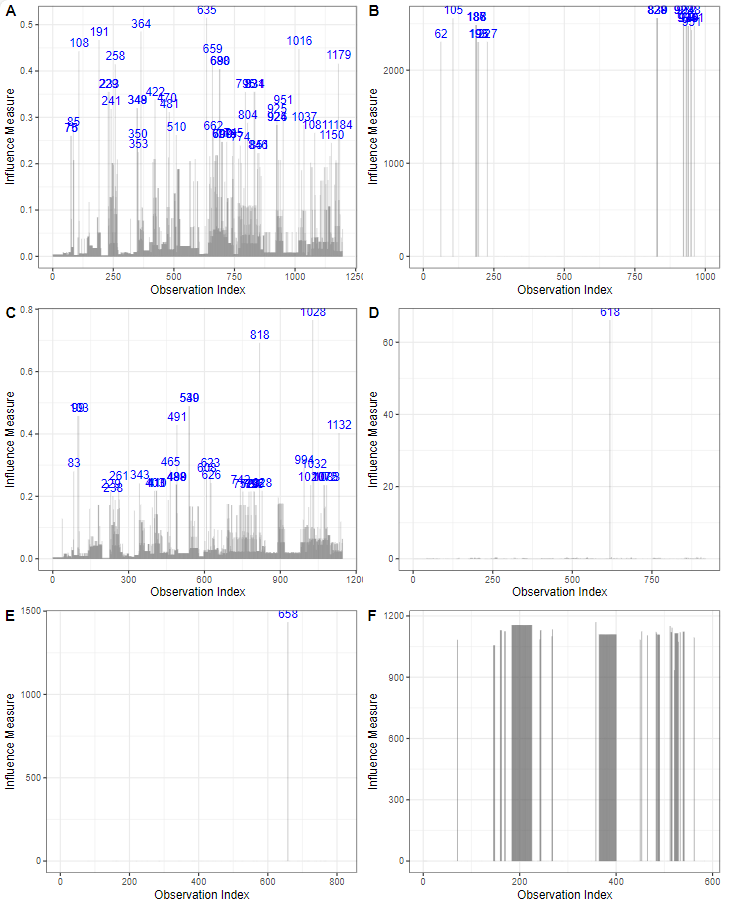


**Figure S3.** Influential case diagnostics for the binomial logistic regression. A) gentamicin; B) clindamycin; C) tetracycline; D) chloramphenicol; E) trimethoprim/sulfamethoxazole; F) enrofloxacin


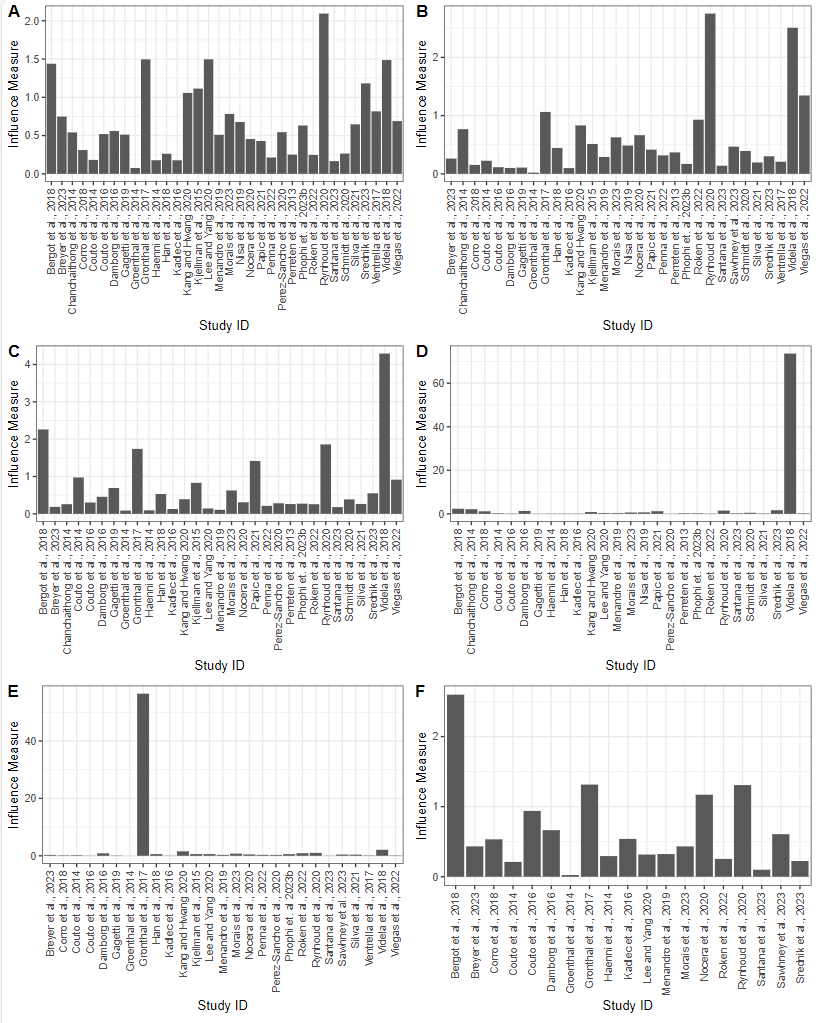


**Figure S4.** Sensitivity analysis for the binomial logistic regression. A) gentamicin; B) clindamycin; C) tetracycline; D) chloramphenicol; E) trimethoprim/sulfamethoxazole; F) enrofloxacin.


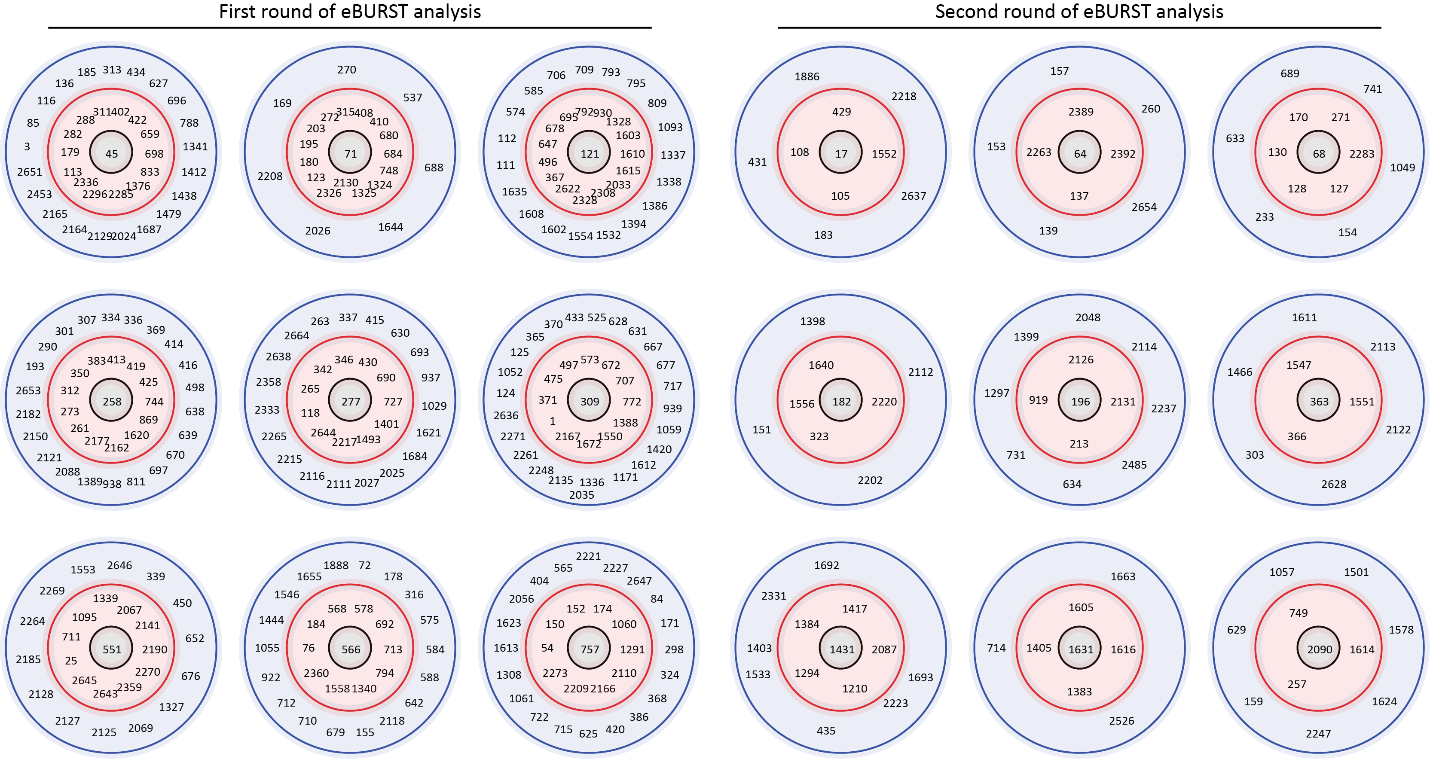


**Figure S5.** eBURST analysis showing clonal complex (CC) clustering of MRSP sequence types (ST). Founder STs are represented the inner circle, while single and double-loci variant STs associated with each CC are provided in the middle end outer circle, respectively.


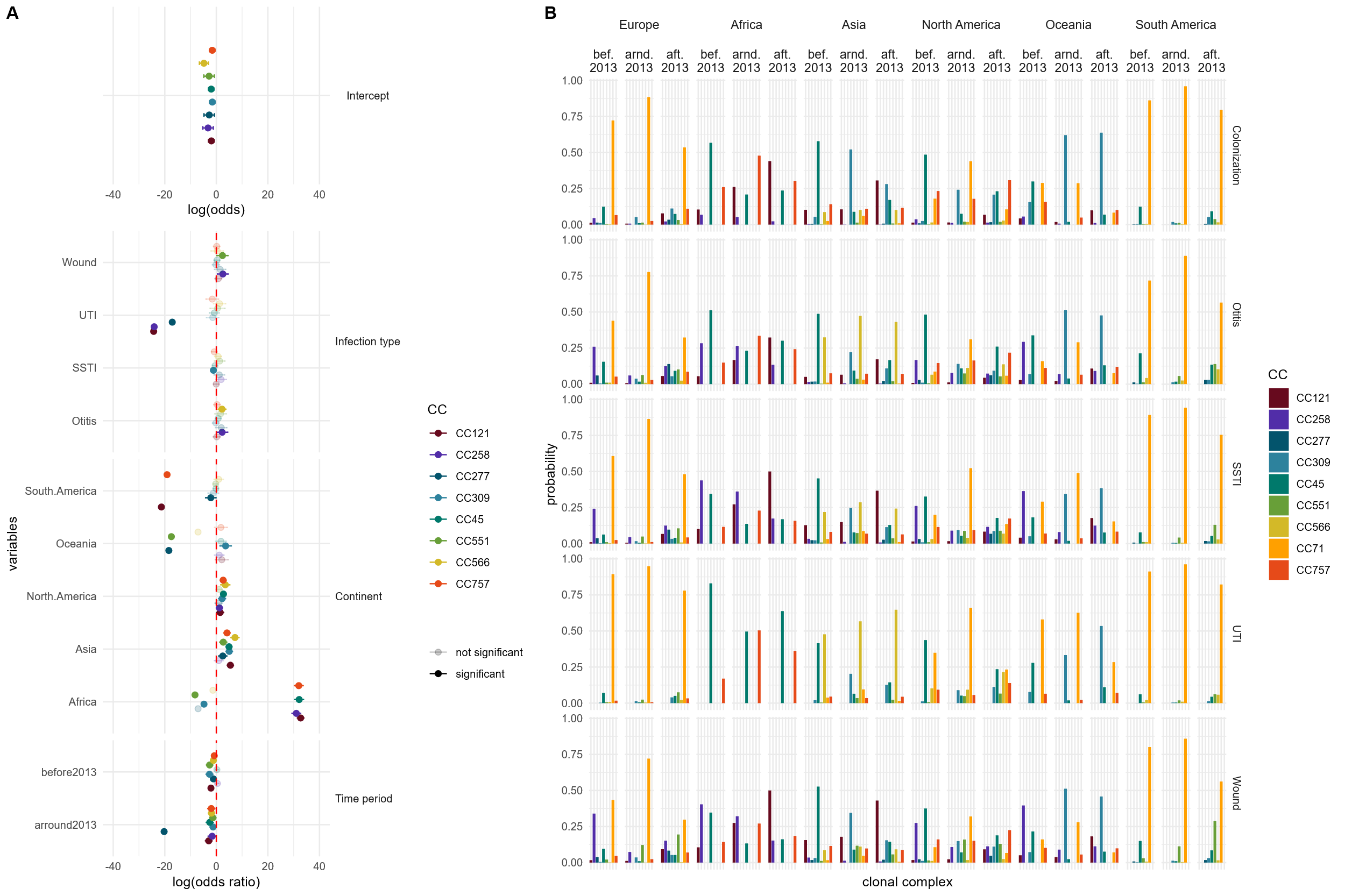


**Figure S6.** Multinomial logistic regression results. A) Association between individual CC and colonization and sample types. Significant odds ratios are shown as filled colors. B) Predicted probability of detecting individual CC in colonization and sample types.
